# SIRT2 Inhibition Results in Meiotic Arrest, Mitochondrial Dysfunction, and Disturbance of Redox Homeostasis during Bovine Oocyte Maturation

**DOI:** 10.3390/ijms20061365

**Published:** 2019-03-18

**Authors:** Dejun Xu, Lin Wu, Xiaohan Jiang, Li Yang, Jianyong Cheng, Huali Chen, Rongmao Hua, Guoxia Geng, Lulu Yang, Qingwang Li

**Affiliations:** 1 College of Animal Science and Technology, Northwest Agriculture and Forestry University, Yangling 712100, China; 2015060124@nwafu.edu.cn (D.X.); nwafu1@sina.com (L.W.); nwafuc@sina.com (X.J.); nwafuj@sina.com (L.Y.); nwafu6@sina.com (J.C.); nwafu3@sina.com (H.C.); esserver@yeah.net (R.H.); 2 College of Veterinary Medicine, Northwest Agriculture and Forestry University, Yangling 712100, China; nwafuh@sina.com (G.G.); nwyll@sina.com (L.Y.)

**Keywords:** SIRT2, oocyte maturation, meiosis, mitochondria, acetylation

## Abstract

SIRT2, a member of the sirtuin family, has been recently shown to exert important effects on mitosis and/or metabolism. However, its roles in oocyte maturation have not been fully clarified. In this study, SIRT2, located in the cytoplasm and nucleus, was found in abundance in the meiotic stage, and its expression gradually decreased until the blastocyst stage. Treatment with SIRT2 inhibitors resulted in the prevention of oocyte maturation and the formation of poor-quality oocytes. By performing confocal scanning and quantitative analysis, the results showed that SIRT2 inhibition induced prominent defects in spindle/chromosome morphology, and led to the hyperacetylation of α-tubulin and H4K16. In particular, SIRT2 inhibition impeded cytoplasmic maturation by disturbing the normal distribution of cortical granules, endoplasmic reticulum, and mitochondria during oocyte meiosis. Meanwhile, exposure to SirReal2 led to elevated intracellular reactive oxygen species (ROS) accumulation, low ATP production, and reduced mitochondrial membrane potential in oocytes. Further analysis revealed that SIRT2 inhibition modulated mitochondrial biogenesis and dynamics via the downregulation of TFAM and Mfn2, and the upregulation of DRP1. Mechanistically, SIRT2 inhibition blocked the nuclear translocation of FoxO3a by increasing FoxO3a acetylation, thereby downregulating the expression of FoxO3a-dependent antioxidant genes *SOD2* and *Cat*. These results provide insights into the potential mechanisms by which SIRT2-dependent deacetylation activity exerts its effects on oocyte quality.

## 1. Introduction

In mammals, oocytes, the largest reproductive stem cells, provide the maternal genetic materials, the majority of organelle, membranes, and cytoplasmic components required for embryo development. In vivo, oocytes are arrested within ovarian follicles at the germinal vesicle (GV) stage. Following stimulation by pituitary luteinizing hormone (LH), fully grown oocytes initiate meiosis. Then, oocytes proceed through the meiosis I (MI) stage, in which microtubules are organized into the spindle and the chromosomes initially separate at the spindle equator. After extruding the first polar body (Pb1), oocytes are arrested at the metaphase II (MII) stage for fertilization [1,2]. However, poor oocyte quality leads to meiotic arrest or infertility. It is well-known that nuclear and cytoplasmic maturation are both required for the successful fertilization of the oocyte, and subsequent embryogenesis [3,4,5]. Before that, oocytes must go through a prolonged and complicated maturation process in vivo [6]. Although in vitro maturation (IVM) has been widely used to produce mature oocytes, the quality and developmental potential of IVM oocytes are much lower than those of oocytes matured in vivo [7,8]. Therefore, clarification of the maturation mechanisms in oocytes is of great importance for the optimization of IVM protocols.

The IVM of an oocyte is a complex process that involves changes in various molecules or organelles, and these processes are controlled by a vast number of intracellular or environmental factors. In particular, higher levels of reactive oxygen species (ROS), which are produced by intrinsic cellular metabolism or which originate from the culture medium, cause oocyte meiotic arrest in vitro [9]. During the meiotic stage, microtubules are organized into the specialized barrel-shaped bipolar spindle, and all of the chromosomes align at the spindle equator [1]. Spindle/chromosome defects give rise to aneuploid embryos when fertilized, resulting in the stagnation of early embryonic development. The accurate regulation of spindle assembly and chromosome segregation is necessary for oocyte nuclear maturation. Unfortunately, the meiotic divisions are prone to error [10,11]. However, the means by which good-quality oocytes attenuate those mistakes in meiosis remain largely unveiled.

Nuclear maturation mainly involves chromosomal segregation, whereas cytoplasmic maturation modulates the redistribution of organelles, including cortical granules (CGs), endoplasmic reticulum (ER), and mitochondria [11], all of which are requires for successful fertilization and embryogenesis. In particular, mitochondria are the organelles that are richest in maternal genetic material, generate the majority of cellular ATP, and provide energy for chromosomal segregation or fertilization in oocytes. It is known that mitochondrial function is a determinant of oocyte maturation [12]. However, the high mitochondrial membrane potential required for ATP synthesis, favors the production of elevated ROS levels [13]. Therefore, the cellular antioxidant system components, such as superoxide dismutase (SOD), catalase (CAT), and glutathione peroxidase (GPx), are important for defending the cell against ROS-induced deleterious effects during IVM [5]. The translocation of CGs to the cell cortex is commonly regarded as an indicator of cytoplasmic maturation [14]. Meanwhile, oocyte cytoplasmic maturation is associated with ER reorganization from a network of cytoplasmic accumulations in the germinal vesicle-stage to a network of distinctive cortical clusters in the MII stage [15]. It has recently been reported that the abnormal distribution of ER results in poor oocyte quality [16].

The sirtuin family comprises nicotinamide adenine dinucleotide (NAD)-dependent deacylases that display diverse functions toward histone or non-histone targets [17,18]. Seven homologous proteins (SIRT1-7) have been observed in mammals, and they vary in substrate specificity, subcellular localization, and biological function [19]. Of these seven, SIRT1 has been the most extensively investigated sirtuin to date, and is known to possess a wide range of substrates. SIRT1, 6, and 7 are primarily reported to reside in the nucleus, whereas SIRT3, 4, and 5 are located in the mitochondria [20,21,22]. Notably, SIRT2 is the only member of the sirtuin family that predominantly exists in the cytoplasm, but it can also be found in the nucleus [23]. Similar to other sirtuin family members, SIRT2 is expressed in a wide variety of tissues, including the brain, muscle, liver, testes, kidney, and adipose tissue [24,25,26]. Previous evidence has suggested that SIRT2 plays roles in the regulation of mitotic progression [27], glucose metabolism [28], oxidative stress response [24], microtubule dynamics [29], and chromatin alignment [30], via the deacetylation of various targets. Although previous studies have shown that SIRT2 is associated with oocyte meiosis in a mouse model [30,31], its functions in oocyte maturation have not been investigated in larger animals. In particular, the underlying mechanisms of SIRT2 that drive oocyte maturation remain largely unveiled.

SirReal2 is a novel and selective SIRT2 inhibitor that directly and potently inhibits the deacetylation activity of SIRT2. This inhibitor is unable to affect the activity of the other class-I sirtuins SIRT1 and SIRT3 in vitro [32], and it shows almost no cytotoxicity at concentrations of <10 μM in several types of cells [33]. Thus, in the present study, bovine oocytes were cultured in IVM medium supplemented with 1, 2, and 5 μM SirReal2, and the parameters of nuclear and cytoplasmic maturation were investigated. Furthermore, we attempted to explore the SIRT2 deacetylation targets and downstream effects during oocyte meiosis that may regulate oocyte maturation. The findings of this study may offer a novel target for improving oocyte quality during IVM.

## 2. Results

### 2.1. SIRT2 is Abundantly Expressed During Oocyte Meiosis

**Figure 1:**
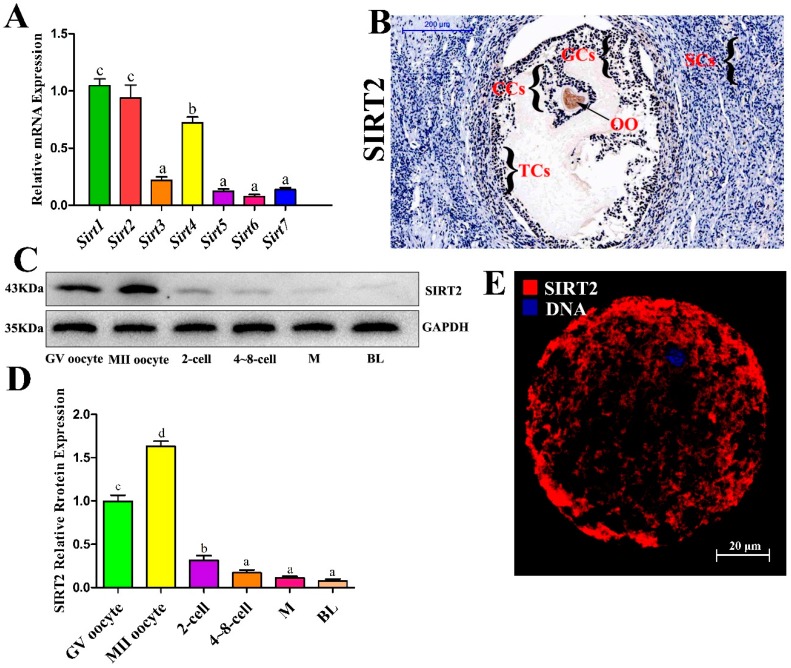
SIRT2 was strongly expressed during oocyte meiosis. (**A**) Sirtuin gene expression in the bovine oocyte. Oocytes in the germinal vesicle (GV) stage were collected for RNA sampling. The messenger RNA (mRNA) levels of *Sir1*, *Sirt2*, *Sirt3*, *Sirt4*, *Sirt5*, *Sirt6*, and *Sirt7* were investigated with RT-PCR analysis. (**B**) The localization of SIRT2 in bovine ovarian cells observed with immunochemistry. Immuno-specific staining was brown, indicating immunopositive cells. Immunohistochemistry was performed on three different slides of ovarian cells from three different bovines. Oocyte, OO; granular cells, GCs; cumulus cells, CCs; theca cells, TCs; Sertoli cells, SCs. Bar: 200 μM. (**C**) The protein expression of SIRT2 during oocyte early development. Each stage of oocyte development is as follows: GV, metaphase II (MII), 2-cell, and approximately 4-to 8-cell, morula (M), and blastocyst (BL) were collected for protein sampling. SIRT2 protein abundance was examined by Western blot analysis. (**D**) Quantitative analysis of SIRT2 protein expression. Band intensities normalized to GAPDH are shown, and data are shown as the means ± SEM of three independent replicates. Bars with different letters (a, b, c, d) indicate significant differences, *p* < 0.05. (**E**) Cellular localization of SIRT2 in oocyte. Oocytes were immunolabeled with anti-SIRT2 antibody (Red) and counterstained with 2-(4-amidinophenyl)-6-indolecarbamidine dihydrochloride (DAPI) to visualize DNA (Blue). Bar: 20 µM.

To investigate the possible involvement of sirtuins in oocytes, the messenger RNA (mRNA) expression levels of *Sirt1-7* were detected by real-time quantitative reverse transcriotion-polymerase chain reaction (RT-qPCR) assays. The data showed that all sirtuin members were present in oocytes, and the expression of *Sirt1*, *Sirt2*, *Sirt4* was higher than that of other members (Figure 1A). As shown in Figure 1B, SIRT2 was found in oocytes, granular cells, cumulus cells, and theca cells, whereas it was rarely observed in Sertoli cells. Furthermore, we first examined the protein expression of SIRT2 in vitro during the early development stage of oocyte by Western blot analysis. SIRT2 was expressed at a high level in the meiotic stage, particularly in the MII oocyte stage (Figure 1C,D). However, after the first cleavage, SIRT2 expression was downregulated until blastocyst stage (Figure 1C,D). By performing confocal scanning, we found that SIRT2 localized in the cytoplasm and nucleus (Figure 1E). These findings reveal that SIRT2 may play important roles in oocyte maturation, and that it weakly functions during embryonic development.

### 2.2. SIRT2 Inhibition Disturbs Meiotic Progression

**Figure 2:**
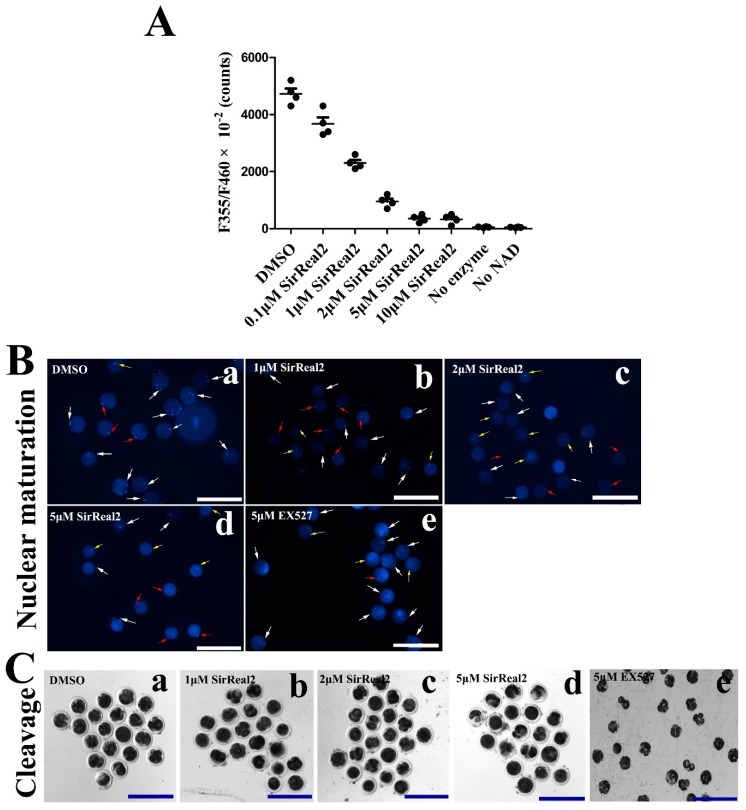
SIRT2 inhibition caused meiotic arrest in vitro. (**A**) Activity analyses of SirReal2-mediated SIRT2 inhibition. The activities of SIRT2 were shown as 355 nm/460 nm × 10^−2^ at concentrations of 0.1, 1, 2, 5, and 10 μM SirReal2. (**B**) Representative images of the effects of SIRT1 or SIRT2 on the nuclear maturation of oocytes. Bovine oocytes were cultured in IVM medium supplemented with or without various concentrations of SirReal2 (b–d), DMSO (a), 5 μM EX527 (e), respectively. Then, the nuclear maturation status was examined with DAPI. GV, MI, and MII oocytes are indicated by yellow, red, and white arrows, respectively. Scale bars: 300 μm. (**C**) Representative images of cleavage embryos in the control (a), 1, 2, 5 μM SirReal2-exposed (b–d), and 5 μM EX527-exposed groups (e), respectively. Scale bars: 300 μm.

As shown in Figure 2A, SIRT2 activities were potently blocked by SirReal2 in a dose-dependent manner, indicating that SirReal2 is an effective SIRT2 inhibitor, and that the concentrations of 1, 2, and 5 μM were suitable for the oocytes in this study. To explore the role of sirtuins in oocyte meiosis, bovine oocytes were treated with either the SIRT1 inhibitor EX527 or the SIRT2 inhibitor SirReal2 during IVM. By performing nuclear staining and quantitative analysis, we found that treatment with SirReal2 resulted in meiotic arrest in a dose-dependent manner (Table 1, Figure 2B). In addition, a significant decrease in cleavage embryos was observed in SirReal2-exposed oocytes, indicating that SIRT2 inhibition led to poor-quality oocytes (Table 1, Figure 2C). Although SIRT1 inhibition also prevented oocyte cleavage, it had almost no effect on nuclear maturation (Table 1; Figure 2B,C). These results indicate that SIRT2 is a main regulator of meiotic progression, but SIRT1 is not.

**Figure 3:**
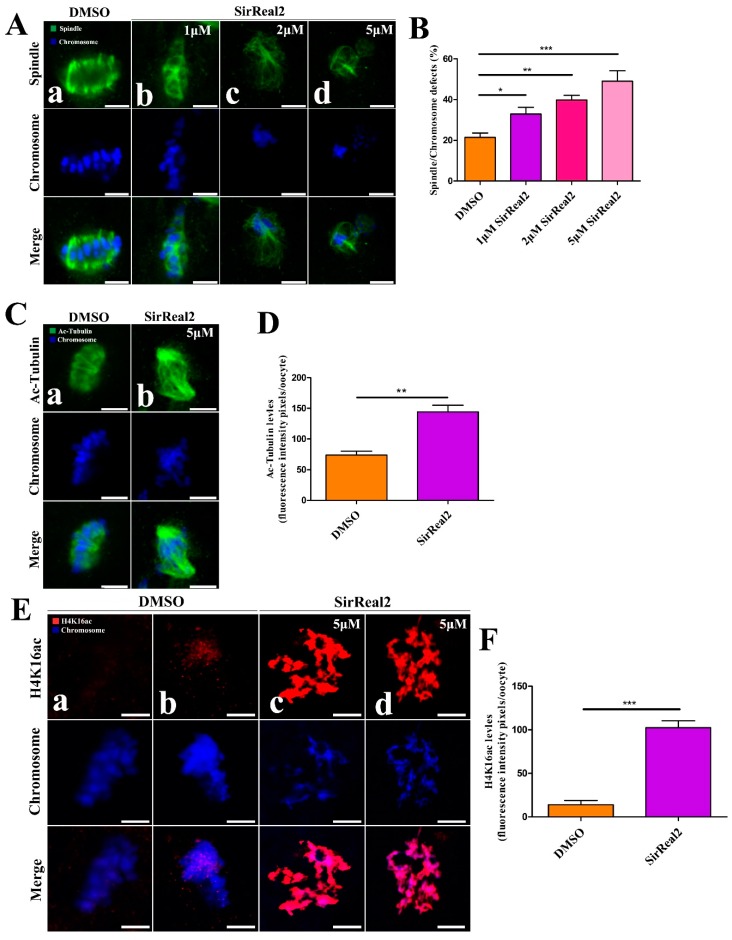
SIRT2 inhibition-induced spindle defects and chromosome misalignment in oocyte meiosis. (**A**) Representative images of spindle morphologies and chromosome alignment in the control (a) and SirReal2-exposed groups (b–d). Oocytes were stained with α-tubulin antibody (green) to visualize the spindles, and counterstained with DAPI (blue) to visualize the chromosomes. Control oocytes presented typical spindle poles and well-aligned chromosomes at the metaphase plate (a), whereas spindle defects and chromosome misalignment were readily observed in SirReal2-exposed oocytes (b–d). Scale bars: 10 μm. (**B**) Quantification of control and SirReal2-exposed groups with spindle/chromosome defects. Oocytes with disorganized spindles and misaligned chromosomes were the result of spindle/chromosome defects. The spindle/chromosome defects in DMSO (*n* = 88), 1 μM SirReal2 (*n* = 87), 2 μM SirReal2 (*n* = 90), 5 μM SirReal2 (*n* = 85) were evaluated, respectively. (**C**) Representative images of acetylated α-tubulin in the control (a) and 5 μM SirReal2-exposed groups (b). Scale bars: 10 μm. (**D**) Quantification of the acetylated α-tubulin fluorescence intensity shown in (**C**). (**E**) Representative images of H4K16ac in the control (a,b) and 5 μM SirReal2-exposed (c,d) oocytes. Oocytes were stained with H4K16ac antibody (red) and counterstained with DAPI to visualize chromosomes (blue). Scale bars: 5 μm. (**F**) Quantification of H4K16ac was analyzed with Image-Pro Plus software. Data are expressed as the mean percentage ± SEM from three independent experiments in which at least 150 oocytes were analyzed. * *p* < 0.05, ** *p* < 0.01, ***p < 0.001, comparing the indicated groups.

To further confirm how SIRT2 mediates oocyte meiosis, oocytes were treated with SirReal2 (1, 2, or 5 μM) for 24 h. They were then immunolabeled with anti-tubulin antibody, to visualize the spindle, and stained with DAPI to visualize the chromosomes. We found that spindle/chromosome defects were readily observed at a high frequency in the groups treated with SirReal2 (Figure 3A,B). To test whether SIRT2 modulates spindle function by directly deacetylating α-tubulin, the acetylation levels of α-tubulin were evaluated by using anti-acetylated α-tubulin antibody. As shown in Figure 3C,D, the levels of acetylated α-tubulin were significantly increased by SIRT2 inhibition. Of note, the hypoacetylation of H4K16 is critical to the maintenance of chromosome segregation in normal conditions. Thus, we examined the levels of H4K16 acetylation in the oocyte after treatment with SirReal2. As expected, SIRT2 inhibition resulted in a drastic increase in H4K16 acetylation (Figure 3E,F). Altogether, SIRT2 inhibition disturbed microtubule dynamics and caused chromosome misalignment by inducing the hyperacetylation of α-tubulin and H4K16, resulting in meiotic defects.

### 2.3. SIRT2 Inhibition Blocks Cytoplasmic Maturation

**Figure 4:**
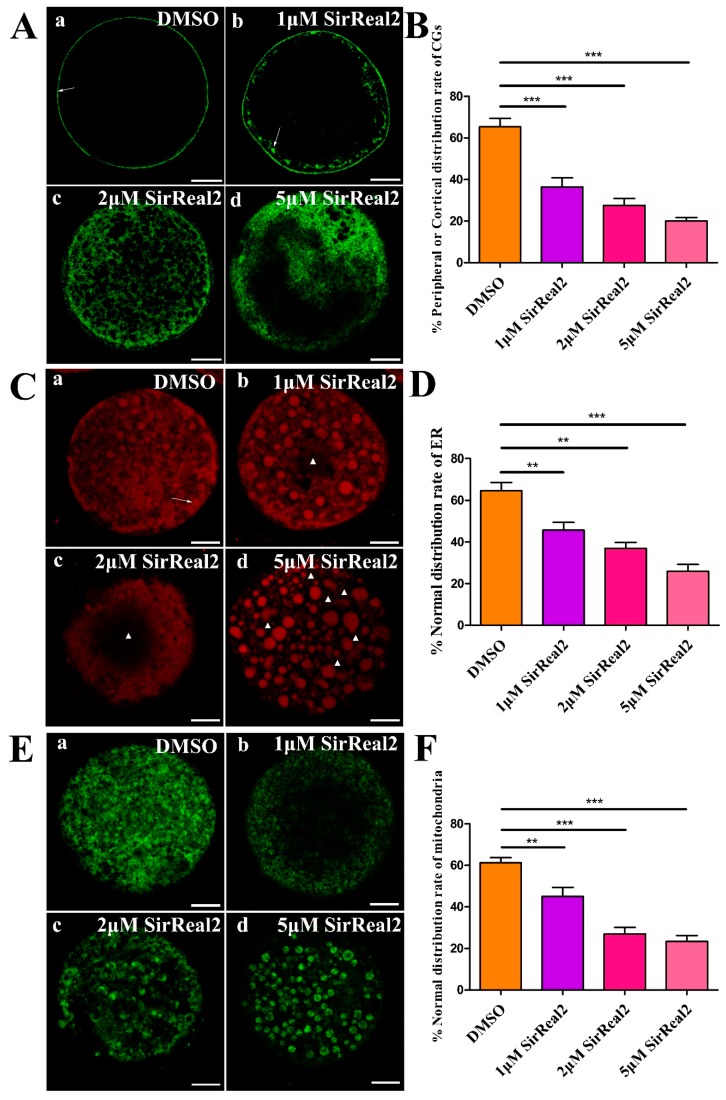
SIRT2 disturbs the redistribution of organelles. (**A**) Representative images of CG distribution patterns visualized with fluorescein isothiocyanate conjugate lectin from peanut (FITC–PNA) in the control group (a) and SirReal2-exposed groups (b–d). (a) Peripheral CG distribution (arrowheads). (b) Cortical distribution (arrowheads). (c) Homogeneous distribution. (d) Nonhomogeneous or abnormal distribution. Scale bar: 20 μm. (**B**) Percentage of peripheral or cortical distribution of GCs in the control and SirReal2-exposed groups. (**C**) Representative images of ER distribution patterns visualized with the ER-tracker red kit in the control group (a) and SirReal2-exposed groups (b–d). (a) Normal distribution of ER. ER was evenly distributed throughout the cytoplasm, with ER clusters in the peripheral area (arrowhead). (b–d) Abnormal distribution of ER: No ER signal was detected in some regions (asterisk) of the cytoplasm. Scale bar: 20 μm. (**D**) Percentage of normal distribution of ER in the control and SirReal2-exposed groups. (**E**) Representative images of mitochondrial distribution patterns visualized with the mito-tracker green in the control group (a) and SirReal2-exposed groups (b–d). (a) Normal distribution. Mitochondria were distributed throughout the cytoplasm. (b–d) Abnormal distribution of mitochondria. No mitochondrial signals were observed in some areas of the cytoplasm. Scale bar: 20 μm. (**F**) Percentage of normal distribution of mitochondria in the control and SirReal2-exposed groups. Data are expressed as the mean percentage ± SEM from three independent experiments in which at least 150 oocytes were analyzed. ** *p* < 0.01, *** *p* < 0.001, comparing the indicated groups.

The intracellular events leading to the redistribution of organelle, including CGs, ER, and mitochondria, are necessary for oocyte cytoplasmic maturation. Therefore, we first examined the functions of SIRT2 during cytoplasmic maturation events with confocal scanning and quantitative analysis. As shown in Figure 4A,B, the peripheral or cortical distribution rates of CGs were obviously reduced by treatment with SirReal2 in a dose-dependent manner. The normal distribution rates of ER and mitochondria were also significantly lower in the control group than in the 1, 2, or 5 μM SirReal2-treated groups (Figure 4C–F). The results indicate that SIRT2 inhibition disturbs oocyte cytoplasmic maturation by regulating various intracellular events.

### 2.4. SIRT2 Inhibition Induces Mitochondrial Dysfunction

**Figure 5:**
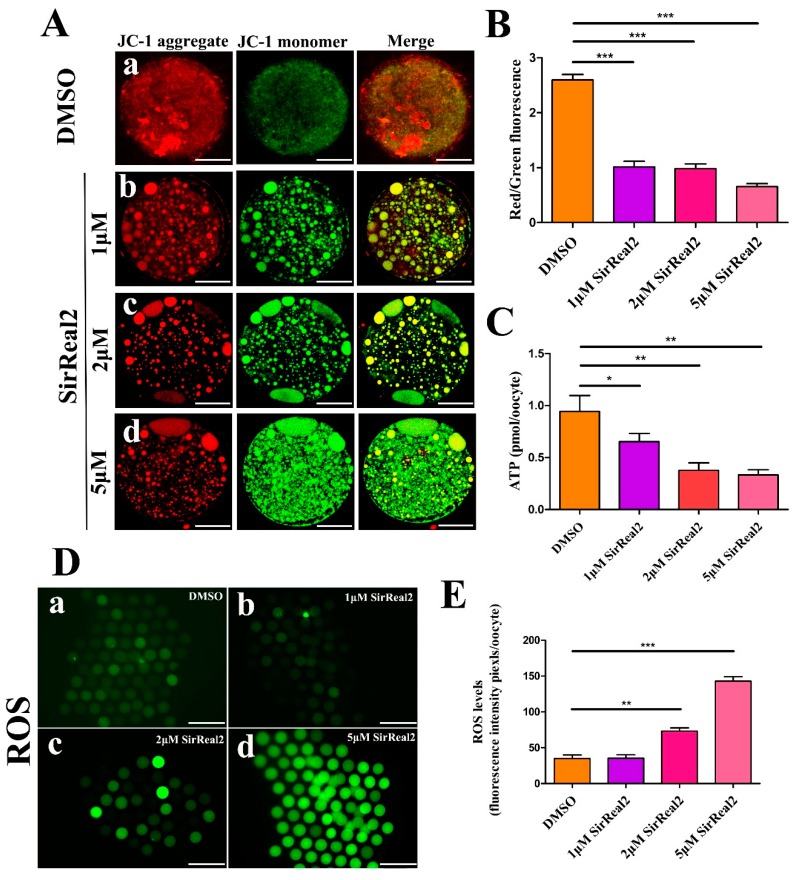
SIRT2 inhibition resulted in mitochondrial dysfunction. (**A**) Representative images of mitochondrial membrane potential in the control group (a) and SirReal2-exposed groups (b–d). JC-1 staining for visualizing the mitochondrial membrane potential of oocytes. The red fluorescence (JC-1 aggregates) indicates high membrane potential, whereas, the green fluorescence (JC-1 monomers) indicates low membrane potential. Scale bar: 30 μm. (**B**) Fluorescence pixel ratio (red/green) in the control and SirReal2-exposed groups. (**C**) Histogram showing the ATP level in the control and SirReal2-exposed groups. (**D**) Representative images of dichloro-dihydro-fluorescein diacetate (DCFH–DA) fluorescence (green) in the control (a) and SirReal2-exposed groups (b–d). Scale bar: 200 μm. (**E**) Quantification of reactive oxygen species (ROS) fluorescence intensity was analyzed with Image-Pro Plus software. Data are expressed as the mean percentage ± SEM from three independent experiments, in which at least 100 oocytes were analyzed. * *p* < 0.05, ** *p* < 0.01, ***p < 0.001, comparing the indicated groups.

We next investigated whether blocking SIRT2 activity affected mitochondrial function. As shown in Figure 5A,B, a significant decline in mitochondrial membrane potential (ΔΨm) was observed in the SirReal2-exposed groups. In addition, SIRT2 inhibition resulted in lower ATP contents compared with the control group; specifically, an approximately 60% reduction in ATP content was observed in the 5 μM SirReal2 groups, indicating impaired mitochondrial function (Figure 5C). Unexpectedly, the intracellular ROS levels were significantly increased by treatment with 2, and 5 μM SirReal2 (Figure 5D,E). This reveals that SIRT2 may depend on an as-yet-unidentified action that modulates the antioxidant system.

**Figure 6:**
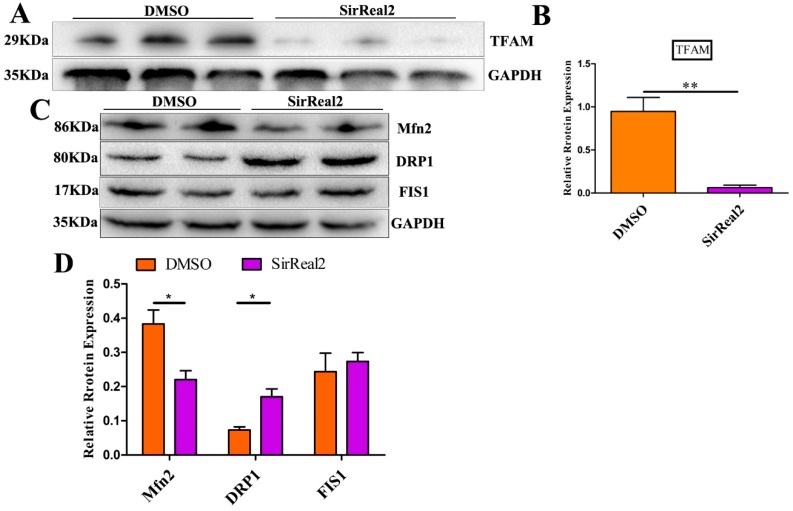
SIRT2 regulated mitochondrial biogenesis and dynamics. (**A**) Western blot analysis revealed reduced mitochondrial transcription factor 1 (TFAM) expression in SirReal2-exposed (5 μM) oocytes. (**B**) Densitometry quantification and statistical analysis of TFAM were revealed using Quantity One software. The ratio of TFAM to GAPDH expression was normalized and the values are indicated. (**C**) Western blot analysis revealed that mitochondrial fission-fusion dynamics were disturbed by SIRT2 inhibition (5 μM SirReal2). (**D**) Densitometry quantification and statistical analysis of Mfn2, DRP1, and FIS1 were revealed. The ratios of Mfn2 to GAPDH, DRP1 to GAPDH, and FIS1 to GAPDH expression were normalized and the values are indicated. Data are shown as the means ± SEM of three independent replicates. * *p* < 0.05, ** *p* < 0.01, comparing the indicated groups.

The specific effect of SIRT2 on mitochondrial function led us to hypothesize that SIRT2 may have a regulatory role in mitochondrial biogenesis and/or dynamics. Thus, after treatment with 5 μM SirReal2 during IVM, the major proteins involved in mitochondrial functions in oocytes were detected with Western blot assays. As shown in Figure 6A,B, a significant decrease in mitochondrial transcription factor A (TFAM) expression was observed in the groups treated with SirReal2. This finding supports the proposal that SIRT2 is required for mitochondrial function by modulating TFAM-dependent mitochondrial biogenesis. We next found that the expression of the mitochondrial fusion-related protein Mfn2 was significantly lower in the control group than in the SirReal2-treated group (Figure 6C,D). Although no significant changes were observed in FIS1, the expression of mitochondrial fission-related protein Drp1 was significantly increased by SIRT2 inhibition. These results suggest that SIRT2 inhibition disturbs the homeostasis of mitochondrial fission–fusion dynamics.

### 2.5. SIRT2 Inhibition Increases Cellular ROS Levels By Blocking the FoxO3a–Sod2/Cat Axis

**Figure 7:**
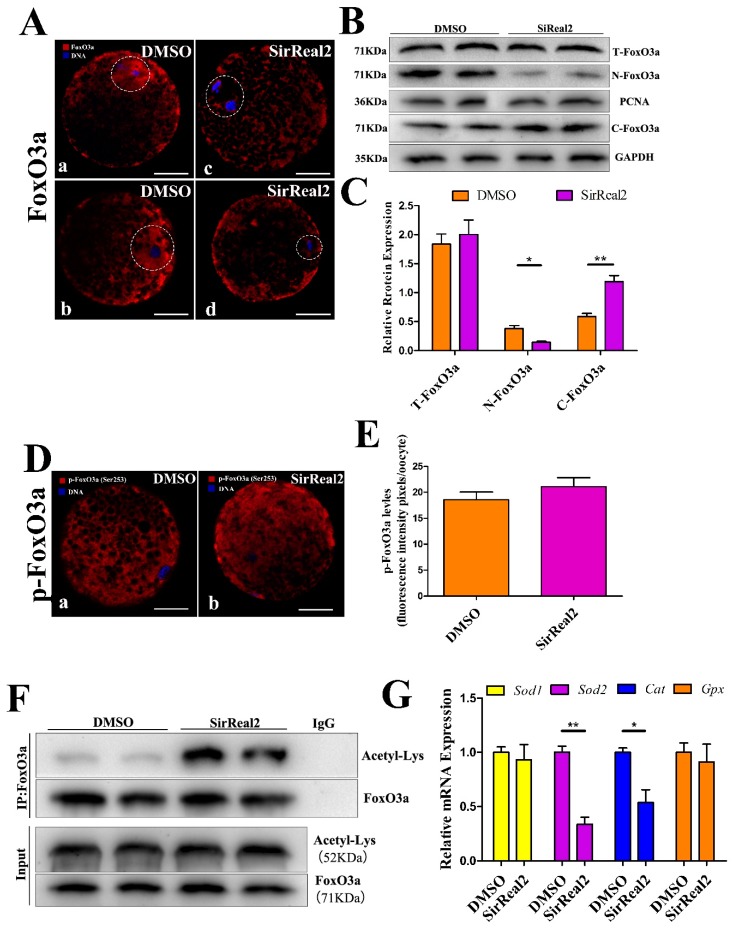
SIRT2 mediated the FoxO3a–SOD2/Cat axis. (**A**) SIRT2 inhibition blocked the nuclear translocation of FoxO3a. Oocytes were immunolabeled with FoxO3a antibody (red) and counterstained with DAPI to visualize DNA (blue) in the control (a,b) and 5 μM SirReal2-exposed groups (c, d). At least 100 control oocytes and 100 SirReal2-exposed oocytes were analyzed. Scale bar: 20 μm. (**B**) The protein expression of FoxO3 in the nucleus, cytoplasm, and overall. GAPDH and proliferating cell nuclear antigen (PCNA) were used as internal controls for the cytoplasmic and nuclear fractions, respectively. (**C**) Quantitative analysis of protein expression of the T-FoxO3a, N-FoxO3a, and C-FoxO3a. Band intensities normalized to GAPDH or PCNA are shown. The ratios of T-FoxO3a to GAPDH, N-FoxO3a to PCNA, and of C-FoxO3a to GAPDH expression were normalized, and the values are indicated. (**D**) Confocal imaging analysis for p-FoxO3a levels in the control (a) and 5 μM SirReal2-exposed groups (b). Oocytes were immunolabeled with p-FoxO3a (Ser253) antibody (red) and counterstained with DAPI to visualize DNA (blue). Scale bar: 20 μm. (**E**) Quantification of p-FoxO3a was analyzed with Image-Pro Plus software. (**F**) SIRT2 inhibition increased the Ac-FoxO3a level. Coimmunoprecipitation for Ac-FoxO3a was performed using the FoxO3a antibody. The immunoprecipitates were analyzed by Western blot with the Acetyl-Lys antibody. IP, immunoprecipitation. (**G**) The mRNA levels of *Sod1*, *Sod2*, *Cat*, and *Gpx* were measured by RT-PCR in the control and SirReal2-exposed groups. The mRNA level of the control groups was arbitrarily set to 1.0, and that of the treatment groups was estimated relative to the control value. Data are shown as the means ± SEM of three independent replicates. * *p* < 0.05, ** *p* < 0.01, comparing the indicated groups.

Previous data from our study showed that SIRT2 inhibition resulted in mitochondrial dysfunction. Although the reduced ΔΨm may generate lower levels of cellular ROS, SIRT2 inhibition elevated ROS accumulation. We thus hypothesized that SIRT2 may mediate the antioxidant system via its deacetylation targets. Then, the effect of SIRT2 on FoxO3a, a major transcription factor of antioxidant genes, was investigated. As shown in Figure 7A, SIRT2 inhibition prevented the nuclear translocation of FoxO3a. The same result was further confirmed by Western blot assays. However, no significant changes were observed in FoxO3a expression between the control group and the SirReal2-treated group (Figure 7B,C).

We next investigated whether SIRT2 affected the subcellular localization of FoxO3a via post-translational modification. For this purpose, the phosphorylated levels of FoxO3a were quantified by immunofluorescence assays with anti-pFoxO3a antibody. Unexpectedly, as shown in Figure 7D,E, SIRT2 inhibition had no effect on p-FoxO3a levels. In addition, we analyzed FoxO3a acetylation levels by performing co-immunoprecipitation assays. The results showed that SIRT2 inhibition increased FoxO3a acetylation (Figure 7F), indicating that SIRT2-dependent deacetylation plays a major role in the nuclear translocation of FoxO3a during oocyte meiosis. Furthermore, the mRNA expression of antioxidant enzymes was examined using RT-qPCR assays. As shown in Figure 7G, the mRNA levels of *SOD2* and *Cat* were obviously reduced by SIRT2 inhibition, whereas there were no significant changes in *SOD1* and *Gpx* mRNA levels. Overall, these results support the previous hypothesis that SIRT2 prevents ROS accumulation by activating the FoxO3a–Sod2/Cat axis during oocyte maturation.

## 3. Discussion

All sirtuins were observed in bovine oocytes, and their expression levels were significantly different in this study. Among them, SIRT1, SIRT2, and SIRT4 were more strongly expressed than other sirtuins. Interestingly, SIRT2 expression was upregulated during the meiotic stage of oocyte development. Similarly, Yumiko et al. have reported that SIRT2 is strongly expressed in the meiotic stage of the mouse oocyte [34]. We found that SIRT1 had almost no effect on oocyte nuclear maturation. In striking contrast, most spindle/chromosome defects were observed in SIRT2 inhibition groups. Thus, we focused on the roles of SIRT2 during oocyte maturation. Although several studies have reported that SIRT2 may regulate oocyte meiosis in a mouse model [30,31], the molecular mechanisms underlying this process remain largely unveiled, especially in the bovine model. Mechanistically, our results suggest that SIRT2 mediate spindle/chromosome organization by deacetylating α-tubulin and histone H4K16. Spindle defects are closely associated with meiotic arrest [35]. Bipolar spindle assembly and function require normal microtubule dynamics [36]. Of note, the enrichment of acetylated α-tubulin is observed in stable microtubules, whereas acetylated α-tubulin is absent from dynamic subcellular structures in eukaryotic cells [37,38]. Previous studies have suggested that tubulin acetylation also influences microtubule stability and function [39,40]. Although acetylated microtubules have been reported in mouse oocytes [30,41], it remains unknown as to how α-tubulin hyperacetylation induces spindle disorganization. On the basis of previous evidence, a possible mechanism is that the SIRT2 inhibition induced-hyperacetylation of tubulin improves microtubule stability, thereby disrupting spindle dynamics for bipolar spindle assembly. Similar to mitosis cells [42], histone H4K16 has been shown to be a substrate of SIRT2 during oocyte meiosis. The acetylation of histone H4 on lysine 16 (H4K16Ac) inhibits the adenosine triphosphate-utilizing chromatin assembly [43]. In addition, H4K16 deacetylation is crucial for chromosome segregation [44]. Thus, these findings support the notion in this study that SIRT2 inhibition results in a misaligned chromosome phenotype by H4K16 hyperacetylation in oocytes. However, our study cannot rule out that SIRT2 might regulate oocyte polarity through GCFD or actin formation during oocyte meiosis. Regrettably, it is difficult to clarify the hypothesis in this study, and future studies will be necessary to assess this hypothesis.

Cytoplasmic maturation is essential for oocyte fertilization and development [45,46]. Here, we revealed that SIRT2 regulates cytoplasmic maturation in bovine oocytes; correspondingly, the abnormal distributions of CGs, ER, and mitochondria were increased in SirReal2-exposed oocytes. Mitochondria are the most prominent organelles in oocytes, and they provide the majority of ATP for essential biosynthesis during oocyte meiosis [47]. It is worth noting that mitochondrial distribution and function are important for oocyte maturation and developmental ability [12,48]. Thus, we focused on how SIRT2-mediated mitochondria function during oocyte meiosis. Compared with SIRT3 and SIRT4, the effect of SIRT2 on mitochondria is not clear, especially in oocytes. Although SIRT2 is not located in mitochondria, here, we revealed that SIRT2 also mediated mitochondrial function in oocytes. Our findings showed that SIRT2 inhibition resulted in the substantial impairment of mitochondrial function. To elucidate the mechanism, the levels of the major proteins involved in mitochondrial functions were investigated. The current results showed that a significant increase in TFAM expression occurred in SirReal2-exposed oocytes. It is well-known that TFAM participates in mitochondrial biogenesis [49], indicating that SIRT2 regulates TFAM-dependent mitochondrial biogenesis. Mitochondrial dynamics is regulated by specific proteins, including Mfn2, DRP1, and FIS1. A recent study reported that Mfn2 deficiency disrupted ER morphology and physical/functional mitochondria–ER interactions during mouse oocyte maturation [50]. Interestingly, a significant decrease in Mfn2 expression and ER normal distribution were observed in SirReal2-exposed oocytes in this study, suggesting that SIRT2 may depend on the Mfn2-mediated regulation of ER distribution. Previous studies have shown that the abnormal expression of Mfn2 and DRP1 changed mitochondrial distribution and ATP production [51,52]. Notably, we found a similar phenomenon, as we observed that mitochondrial redistribution was associated with the expression of Mfn2 and DRP1 in oocytes. Our results revealed that SIRT2 inhibition regulated fission–fusion events by upregulating DRP1 and downregulating Mfn2, thereby disturbing mitochondrial dynamics during oocyte maturation. Similarly, a recent study reported that SIRT2 attenuated ROS-induced mitochondrial dysfunction by mediating the expression of TFAM, Mfn2, and Drp1 in hepatocytes [53]. These findings support the notion that SIRT2 is necessary for cytoplasmic maturation via the modulation of mitochondrial biogenesis and dynamics.

Mitochondrial membrane potential is essential for ATP generation, but a high potential membrane results in elevated ROS [13]. It is worth noting that elevated ROS accumulation, low ATP content, and decreased antioxidant enzyme expression were observed in SirReal2-exposed oocytes in this study. The results suggest that SIRT2 may regulate the antioxidant system by acting on other molecular pathways. Previous evidence has demonstrated that FoxO3a binds to sites in the promoters of SOD2 and catalase, and that it directly induces the expression of these enzymes [24,54,55]. In addition, FoxO3a has been reported to be an SIRT2 deacetylation target [56,57,58]. According to these findings, we anticipated that SIRT2 may depend on FoxO3a to mediate the antioxidant system. We found that SIRT2 inhibition blocked FoxO3a nuclear translocation in oocytes during the meiotic stage. FoxO activity is regulated by post-translational modifications that affect primarily its subcellular localization [59]. Phosphorylation is an important mechanism for the regulation of the subcellular localization and activity of FoxOs [60]. Surprisingly, SIRT2 inhibition had no influence on the phosphorylated levels of FoxO3a. SIRT1 and SIRT3 are known to mediate FoxO3a phosphorylation via Akt [61,62]; here, SIRT2 did not seem to regulate the Akt-dependent FoxO3a phosphorylation pathways in oocytes. However, a second regulatory layer is FoxO acetylation by p300, CBP, and PCAF [63,64,65], followed by deacetylation by class I and II histone deacetylases, including SIRT1 [66,67]. A study showed that the SIRT1 activator resveratrol can promote FoxO1 deacetylation and nuclear retention, thus increasing its activity [68]. Moreover, SIRT3-induced FOXO3a deacetylation resulted in increased nuclear localization and transcriptional activity of FOXO3a under oxidative stress in mice [69]. On the basis of previous evidence, we speculated that a similar mechanism is likely to be present for FoxO3a. Therefore, we attempted to detect the acetylation levels of FoxO3a, using co-immunoprecipitation (co-IP) assays in oocytes. As expected, a significant increase in FoxO3a acetylation was observed in SirReal2-exposed oocytes, indicating that FoxO3a is a substrate of SIRT2. The result is consistent with a previous finding that SIRT2 deacetylates FoxO3a, and increases its transactivation activity in HEK293T cells [56]. In addition, a recent report also showed that SIRT2 drives FoxO3a nuclear translocation by its deacetylation activation in rat kidneys [70]. These findings support our previous results that SIRT2-mediated FOXO3a deacetylation promotes the nuclear transposition and activation of FoxO3a, followed by an increase in *Sod2* and *Cat*.

In summary, our results highlight the important function of SIRT2 in oocyte maturation in vitro. Mechanistically, SIRT2 was found to mediate spindle organization and chromosome alignment, mitochondrial function, and the antioxidant system, by acting on specific targets, including α-tubulin, H4K16, TFAM, and FoxO3a. These findings indicate that SIRT2 activators may offer novel opportunities for improving the oocyte quality during the meiotic stage.

## 4. Materials and Methods

### 4.1. Reagents and Ethics Statement

SirReal2 (Cat#: S7845) and EX527 (Cat#: S1541) were purchased from Selleck chemicals (Houston, TX, USA). Rabbit polyclonal anti-SIRT2 (Cat#: 19655-1-AP), mouse monoclonal anti-α-tubulin (Cat#: 66031-1-Ig), mouse monoclonal anti-acetylated tubulin (Lys40, Cat#: 66200-1-Ig) antibodies were purchased from Proteintech Group Inc. (Wuhan, China). Rabbit polyclonal anti-pFoxO3a (Phospho-Ser253; Cat#: 12199), and rabbit polyclonal anti-FIS1 (Cat#: 33067) antibodies were obtained from Signalway antibody LLC (College park, MD, USA). Rabbit polyclonal anti-TFAM (Cat#: OM108083), rabbit polyclonal anti-Mfn2 (Cat#: OM110904), rabbit polyclonal anti-DRP1 (Cat#: OM250563), and rabbit polyclonal anti-PCNA (Cat#: OM287574) antibodies were purchased from Omnimabs (Alhambra, CA, USA). Rabbit monoclonal anti-GAPDH (Cat#: ab181603), rabbit monoclonal anti-acetyl lysine (Cat#: ab190479), rabbit monoclonal anti-Histone H4 (acetyl K16; ab109463), and rabbit polyclonal anti-PCNA (Cat#: ab70315) antibodies were purchased from Abcam (cambridge, UK). Unless otherwise indicated, the other chemicals and media were purchased from Sigma-Aldrich (St. Louis, MO, USA).

### 4.2. Ethics Statement

The study was performed in accordance with the requirements of the Institutional Animal Care and Use Committee of Northwest Agriculture and Forestry University (Shaanxi, China), and the study of this project (No. IACUC-20180325-16) was approved in 25 March 2018.

### 4.3. Assessment of SIRT2 Activity for SIRT2 Inhibitor

To screen the effective concentrations of SIRT2 inhibitors in oocytes, a dose-response test of SirReal2′s effect on SIRT2 activity was performed with the SIRT2 Activity Assay Kit (Fluorometric, Cat#: ab156066, Abcam, cambridge, UK). Bovine ovaries were obtained from the local abattoir (Shaanxi, China), and immediately transported to the laboratory within 8 h in phosphate-buffered saline (PBS) containing penicillin (100 IU/mL) and streptomycin (100 mg/mL) at 27–30 °C. The cumulus-enclosed oocytes were collected from the follicle, and cumulus cells were removed by hyaluronidase. Then, a pool of 100 oocytes were lysed with 20 μL of lysis buffer at 4 °C, and supplemented with or without various concentrations of SirReal2 (0.1, 1, 2, 5, 10 μM). Briefly, the test compound was added to the samples according to the kit’s instructions, and then incubated for 60 min at room temperature. The fluorescence intensity was read using a luminometer (BioTek, Winooski, VT, USA) with excitation at 355 nm and emission at 460 nm. SIRT2 activity was calculated with F355 nm/460 nm × 10^−2^ (counts).

### 4.4. IVM and Treatment of Bovine Oocytes

Oocytes at a minimum of three layers of cumulus cells were collected for IVM. After washing three times at least with medium 199 (Gibco, Waltham, MA, USA) containing 5% (*v*/*v*) fetal bovine serum (FBS), a group of 50 cumulus-oocyte complexes (COCs) was matured for 24 h in 750 μL of IVM medium at 38.5 °C in 5% CO_2_. The IVM medium consisted of M199 supplemented with 10% (*v*/*v*) FBS, 1% (*v*/*v*) ITS (contains 1.0 mg/mL recombinant human insulin, 0.55 mg/mL human transferrin, and 0.5 μg/mL sodium selenite), 0.1 IU/mL human menopausal gonadotropin, 0.2 mM sodium pyruvate, 10 ng/mL epidermal growth factor, 0.2 IU/mL follicle-stimulating hormone (Ningbo Second Hormone Factory, Ningbo, China), 2 μg/mL 17β-estradiol, 100 IU/mL penicillin, and 100 mg/mL streptomycin. Oocytes were cultured for 24 h in IVM medium supplemented with or without various concentrations of SIRT2 inhibitors (1, 2, 5 μM SirReal2), respectively. The concentrations and incubation periods of SirReal2 used in this study were empirically determined and used as described previously [32,33,71,72].

### 4.5. IVF and Embryo Culture

In vitro fertilization (IVF) was performed in this study according to a previously described procedure, with some modifications [73]. Briefly, after swimming-up for 30 min in IVF medium (Caisson, Smithfield, UT, USA) containing 50 μg/mL heparin and 2.5 mM caffeine, the supernatant bovine spermatozoa were centrifuged at 500× *g* for 10 min. A 50 μL sperm suspension (at a concentration of 2 × 10^6^/mL) was added to about 25 oocytes without cumulus cells in a 50 μL microdrop of IVF medium. After 6 h of IVF at 38.5 °C in 5% CO_2_, a group of 20 oocytes were cultured in a 100 μL microdrop of mSOFaa [74] at 38.5 °C in 5% CO_2_ for seven days, with 70% of the medium being replaced on day 3.

### 4.6. Assessment of Nuclear Maturation Status

After undergoing IVM for 24 h, cumulus cells were stripped in 0.1% (*w*/*v*) hyaluronidase. After being fixed with 4% paraformaldehyde for 10 min, the denuded oocytes were stained with 2-(4-amidinophenyl)-6-indolecarbamidine dihydrochloride (DAPI) for 5 min. Then, the nuclear status of oocytes was observed under a fluorescence microscope (Nikon, Tokyo, Japan). In accordance with a previously described method [75], oocytes with brightly condensed chromatin were regarded as being in the GV phase; oocytes with chromatids gathered at the metaphase plate were classified as MI oocytes; those with two bright spots of chromatin were judged to be in the MII phase, indicating nuclear maturation.

### 4.7. Staining of Mitochondria, CGs, and ER

For mitochondrial staining, oocytes were incubated in M199 containing 100 nM Mito-Tracker Green (Beyotime, Shanghai, China) in the dark at 38.5 °C for 30 min. After washing with M199 containing 0.1% BSA, oocytes were observed with a confocal microscope (Leica, Solms, Germany), and recorded according to a previously described method [12].

For CG staining, zona pellucidae (ZP) were removed from oocytes by treatment with 0.5% (*w*/*v*) pronase for 1 min. After being fixed with 4% paraformaldehyde for 30 min, the ZP-free oocytes were blocked in PBS containing 0.3% (*w*/*v*) BSA for 1 h, and permeabilized with 0.1% (*v*/*v*) Triton X-100 for 5 min. Then, oocytes were incubated in 10 mM FITC conjugated to lectin from *Arachis hypogaea* for 30 min. After washing three times in PBS (5 min per wash), the labeled oocytes were quantified, and CG distribution was observed with a confocal microscope (Leica, Germany). According to a previously described method [76], the distribution of CGs was classified into four groups as follows: (a) peripheral distribution: CGs were adjacent to the plasma membrane, indicating cytoplasmic maturation; (b) cortical distribution: the majority of CGs were spread in the cortical area, indicating partial cytoplasmic maturation; (c) homogeneous distribution: CGs were distributed throughout the cytoplasm, indicating no cytoplasmic maturation; (d) nonhomogeneous or abnormal distribution: anomalous distribution of CGs, indicating poor-quality or degenerated oocytes.

For ER staining, oocytes were labeled with the ER-Tracker Red kit (Beyotime, China). According to the instructions, oocytes were incubated in the ER-Tracker Red medium for 30 min at 37 °C. After washing three times in PBS (5 min each wash), the stained oocytes were quantified, and observed with a confocal microscope (Leica, Germany).

### 4.8. Determination of Intracellular ROS

The intracellular ROS content was detected using the fluorescent dye 2′,7′-dichlorodihydrofluorescein diacetate (DCFH-DA; Beyotime, China) in oocytes. Briefly, oocytes were incubated in 10 μM DCFH-DA for 30 min at 37 °C in the dark. After washing three times with M199 contained 0.1% (*w*/*v*) BSA, the labeled oocytes were quantified, and observed using a fluorescence microscope (Nikon, JP) with 460 nm UV filters. Finally, the fluorescence intensities of the oocytes were analyzed using an Image-Pro Plus 6.0 software (Media Cybernetics, Rockville, MD, USA).

### 4.9. Determination of Intracellular ATP and ΔΨm

After IVM, the ATP content of oocytes was quantified using an ATP-dependent luciferin–luciferase bioluminescence assay (ATP assay kit; Sigma, Shanghai, China), which has a range of 20–1000 pmole/well for the fluorometric assay. Briefly, a pool of 100 denuded oocytes was lysed in 20 μL of cell lysis reagent. Following the kit’s instructions, the standard solutions (0, 1.0, 2.5, 5.0, 7.5, and 10 pmol of ATP; 20 μL) combined with samples (20 μL) were separately added to each well of the 96-well plates supplemented with 100 μL of ATP detection solution, respectively, and luminescence intensity was immediately measured using a luminometer (BioTek, USA). Subsequently, a six-point standard curve (0, 1.0, 2.5, 5.0, 7.5, and 10 pmol of ATP) was obtained for each series of analyses. Finally, the ATP content of the samples was calculated using the formula derived from the standard curve.

For evaluating the mitochondrial membrane potential (ΔΨm), the (ΔΨm) assay kit with JC-1 (Beyotime, China) was used. The procedure was performed according to the kit’s instructions. Briefly, oocytes were incubated in working solution containing 10 μM JC-1 at 38.5 °C for 30 min in the dark. After washing with JC-1 buffer solution, oocytes were observed under a Leica confocal microscope with the same scan settings for each sample. JC-1 aggregates (red fluorescence) were detected with the TRITC channel, which indicated high membrane potentials, whereas JC-1 monomers (green fluorescence) were detected with the FITC channel, which indicated low membrane potentials. The ratio of aggregates (red fluorescence) to monomers (green fluorescence) was calculated to quantify changes in mitochondrial membrane potential. The fluorescence intensities of oocytes were analyzed by Image-Pro Plus 6.0 software.

### 4.10. Quantitative Real-Time PCR for Candidate Genes

A pool of 50 oocytes were added to 6 μL of Lysis buffer (5 mmol/mL dithiothreitol, 20 U/mol RNase inhibitor, 1% NP-40). Total RNA was extracted by repeated pipetting on ice for 20 min. According to the instruction procedure, the first-strand complementary DNA (cDNA) was synthesized with a PrimeScript TM RT Reagent Kit with genomic DNA Eraser (Takara, Kusatsu, JP) on ice. After reversing transcription of the total RNA, the targeted cDNA was performed using CFX96TM Real-Time PCR (Bio-Rad, Hercules, CA, USA) with the SYBR Premix Ex TaqII (2×) Reagent Kit (Takara, JP). The bovine-specific primers for target genes for real-time PCR were listed in Table S1. The thermal cycling parameters for Real-Time PCR were performed in our previously described protocol [72]. Based on the threshold cycle (CT) value of each gene, the mRNA expression was calculated by using the ΔΔ*C*t method. The *GAPDH* gene was used as a housekeeping gene for each sample, and the results are represented as the target gene ratio to *GAPDH* levels.

### 4.11. Western Blotting and Immunoprecipitation

For immunoblotting, a pool of 50 oocytes were lysed in 20 μL of cold radio immunoprecipitation assay (RIPA) buffer (Beyotime, China), containing 50 mM Tris pH 7.4, 150 mM NaCl, 1% Triton X-100, 1% sodium deoxycholate, 0.1% SDS, and 1 mM phenylmethanesulfonyl fluoride (PMSF), the nuclear and cytoplasmic proteins of 200 oocytes from each group were harvested using a Nuclear and Cytoplasmic Protein Extraction Kit (Beyotime, China). Then, each lysed sample was mixed with 5 μL of 5× SDS-PAGE sample loading buffer, and boiled for 5 min at 100 °C. A volume of 25 μL of the lysates of each sample was subjected to 10% SDS-PAGE, and transferred to 0.2 μm polyvinyl fluoride (PVDF) membranes (Beyotime, China). Non-specific binding sites were blocked with a solution of TBST (20 mM Tris-HCl, 150 mM NaCl, 0.05% Tween 20), containing 5% (*w*/*v*) non-fat dry milk, the membranes were incubated in this blocking solution with slight shaking for 60 min at room temperature. Then, the membranes were incubated with primary antibodies (anti-SIRT2, 1:500; anti-TFAM, 1:500; anti-Mfn2, 1:500; anti-DRP1, 1:500; anti-FIS1, 1:500; anti-FoxO3a, 1:500; anti-PCNA, 1:1000; anti-acetyl lysine, 1:500 anti-GAPDH, 1:10,000) overnight at 4 °C. After three washes (10 min per wash) in TBST, the membranes were incubated with slight shaking for 1 h at room temperature with HRP-conjugated secondary antibodies (Sungene Biotech, Tianjin, China 1:5000). After washing, the protein bands were exposed to X-ray film for visualization with ECL Plus (Millipore, Burlington, MA, USA), and the intensities were measured to determine protein abundance with Quantity One software (v. 4.52; Bio-Rad Laboratories).

For immunoprecipitation, 400 denuded oocytes from each treatment group were lysed with 200 μL of cold RIPA buffer. The 10% lysates were analyzed by Western blotting. Non-specific background was eliminated with protein A agarose and normal rabbit IgG (Beyotime, China). Then, anti-FoxO3a antibodies (1:50) were added to the lysate samples, and incubation occurred overnight at 4 °C with slight shaking. The immune complexes were captured by 20 μL protein A agarose (Beyotime, China) with slight shaking for 3 h at 4 °C Also, the IPKine™ HRP-mouse anti-rabbit IgG LCS (Abbkine, Wuhan, China; 1:5000) was used to clear heavy chain blotting contamination from the immunoprecipitation. Then, Western blot analysis was performed for FoxO3a and acetyl-lysine, as previously described, respectively.

### 4.12. Immunohistochemistry

Bovine ovaries were fixed in 4% paraformaldehyde overnight at 4 °C, and then transferred to 70% ethanol. Ovaries were embedded with paraffin, and cut into 5 µm sections for immunostaining. The sections were dewaxed and then dehydrated with sodium citrate buffer (10 mM sodium citrate, 0.05% Tween 20, pH 6) for antigen retrieval. After blocking for 1 h at room temperature in TBST containing 5% normal goat serum (Cell signaling, Boston, MA, USA), the sections were incubated with anti-SIRT2 (1:200), overnight at 4 °C. Negative controls were incubated with nonimmune rabbit IgG (1:200). The sections were washed three times (5 min each wash) before incubation with anti-rabbit secondary antibodies (Beyotime, China). Next, the samples were stained at room temperature by SignalStain^®^ Boost IHC Detection Reagent (Cell signaling, USA). After washing, the sections were counterstained in hematoxylin for 20 s. Images of the stained sections were captured by a Nikon DS-Ri1 digital camera (Nikon, Tokyo, JP).

### 4.13. Immunofluorescence Staining

After being fixed with 4% paraformaldehyde, oocytes were permeabilized in PBS containing 0.5% (*v*/*v*) Triton X-100 for 60 min at room temperature. The permeabilized oocytes were blocked for 1 h with QuickBlock™ blocking buffer (Beyotime, China) at room temperature, and incubated separately with anti-SIRT2 polyclonal antibody (1:100), anti-α-tubulin monoclonal antibody (1:100), anti-acetylated tubulin monoclonal antibody (1:50), anti-H4K16ac polyclonal antibody (1:100), anti-FoxO3a polyclonal antibody (1:100), and anti-pFoxO3a polyclonal antibody (1:50) at 4 °C overnight. After washing three times with PBS-PVA solution, Alexa Fluor 488-labeled goat anti-mouse IgG (1:200; Beyotime, China) was used to visualize the mouse antibodies, and the Alexa Fluor 555-labeled donkey anti-rabbit IgG (1:200; Beyotime, China) was used to visualize rabbit antibodies. Then, the chromosomes were counterstained with DAPI. Next, the stained oocytes were mounted and observed with a confocal microscope (Leica, Germany), and the fluorescence intensities of the stained oocytes were analyzed by Image-Pro Plus 6.0 software (Media Cybernetics, Rockville, MD, USA).

### 4.14. Statistical Analysis

All experiments were biologically repeated at least three times. The data were represented as mean ± standard error of the mean (SEM). Statistical comparisons were analyzed by one-way analysis of variance (ANOVA) by Duncan’s test, and differences between treatment groups were assessed with the *t*-test using SPSS 20.0 statistical software (SPSS, Chicago, IL, USA). A *p* value < 0.05 was considered as a statistically significant difference.

## 5. Conclusions

The present study provides the first direct evidence that SIRT2 mediates oocyte nuclear maturation and cytoplasmic maturation by acting on specific targets. First, we found that SIRT2 inhibition induced the hyperacetylation of α-tubulin and H4K16, thereby causing spindle/chromosome defects. Second, cytoplasmic maturation parameters, including the redistribution of CGs, ER, and mitochondria, were disturbed by SIRT2 inhibition. Third, blocking SIRT2 activity resulted in mitochondrial dysfunction by the downregulation of mtDNA-associated protein TFAM and mitochondrial fusion-related protein Mfn2, and the upregulation of fission-related protein DRP1. Finally, we revealed that SIRT2 regulated ROS levels by activating the FoxO3a–Sod2/Cat axis. Taken together, these results suggest that SIRT2-dependent deacetylation activity is necessary for oocyte maturation.

## Figures and Tables

**Table 1:** The effects of SirReal2 or EX527 on oocyte maturation and development.

Treatment	No. of GV Oocytes (%)	No. of MI Oocytes (%)	No. of MII Oocytes (%)	No. of Cleavage Embryos (%)
DMSO	9 (4.47 ± 0.95) ^a^	26 (12.58 ± 2.95) ^a^	168 (83.01 ± 3.30%) ^c^	106 (70.67 ± 8.08%) ^d^
1 μM SirReal2	40 (19.16 ± 2.90) ^b^	81 (39.45 ± 7.09) ^b^	86 (41.40 ± 3.39%) ^b^	48 (32.00 ± 4.00%) ^b^
2 μM SirReal2	59 (27.74 ± 4.70) ^c^	88 (41.24 ± 7.92) ^b^	66 (31.02 ± 3.26%) ^a^	40 (26.67 ± 3.06%) ^ab^
5 μM SirReal2	78 (37.14 ± 8.19) ^d^	77 (36.27 ± 3.91) ^b^	57 (26.87 ± 1.67%) ^a^	28 (18.67 ± 3.06%) ^a^
5 μM EX527	11 (5.30 ± 1.05) ^a^	33 (15.73 ± 3.22) ^a^	165 (79.11 ± 4.59%) ^c^	83 (55.33 ± 5.03) ^c^

Data are expressed as the mean percentage ± SEM from three independent experiments. Values within a row with different letters (^a^, ^b^, ^c^, ^d^) indicate significant differences (*p* < 0.05).

## Supplementary Materials

Supplementary materials can be found at https://www.mdpi.com/1422-0067/20/6/1365/s1. Table S1. Sequences for primers used in quantitative real-time RT-PCR.
