# Supplementary material for: SIRT2 Inhibition Results in Meiotic Arrest, Mitochondrial Dysfunction, and Disturbance of Redox Homeostasis during Bovine Oocyte Maturation
Source: Int J Mol Sci. 2019 Mar 18;20(6):1365. doi: 10.3390/ijms20061365 (PMC6472277; doi:10.3390/ijms20061365)
Supplement: Supplementary file 1 [file ijms-20-01365-s001.pdf]

Table S1. Sequences for primers used in quantitative real-time RT-PCR.

Gene Name	Primer Sequences (5'–3')	GenBank Accession No.
SIRT1	F:GGAGCAGATTAGTAAACGCCT<br>R:CTTTCATCCTCCATGGGTTC	NM_001192980
SIRT2	F:CAACCTGGAGAAATACCGTCTT<br>R:CAGTCCTTTTTCCTTCAGCAG	NM_001113531.1
SIRT3	F:GCATGGCGTTGTTTCCTCGT<br>R:TGTCACTTGAGGCACCAGCA	NM_001206669.1
SIRT4	F:CCCCGCTTCCTCTATCTGA<br>R:GGGCTCTTTCTTCTGCCTAAT	NM_001075785.1
SIRT5	F:ACTTCCTCCGTGGTCTATCC<br>R:GACTTTCCAGTATTTGGCTCAC	NM_001034295.2
SIRT6	F:GCGGTCTACCCGAGGTCTTC<br>R:ACACCACACTGGAGGACTGC	NM_001098084.1
SIRT7	F:AGCCTCTATCCCAGATTACCG<br>R:ACACCCGCACATATTCCCTA	NM_001075217.1
Cat	F:AGATGGACACAGGCACATGA<br>R:ATTGAAAAGATCGCGGAGGC	NM_001035386.2
Sod1	F:CATCCACTTCGAGGCAAAGG<br>R:CATCCACTTCGAGGCAAAGG	NM_174615
Sod2	F:GGACGCTTACAGATTGCTGC<br>R:ATGCTCCCACACGTCAATCC	NM_201527.2
Gpx	F:ACCATCTATGAGTACGGGGC<br>R: GCAGTGCATTCAGTTCAACG	NM_174077.5
GAPDH	F:CACCCTCAAGATTGTCAGCA<br>R:GGTCATAAGTCCCTCCACGA	NM_001034034
